# Supplementary figures and images for: Predicting time to graduation at a large enrollment American university
Source: PLoS One. 2020 Nov 13;15(11):e0242334. doi: 10.1371/journal.pone.0242334 (PMC7665823; doi:10.1371/journal.pone.0242334)

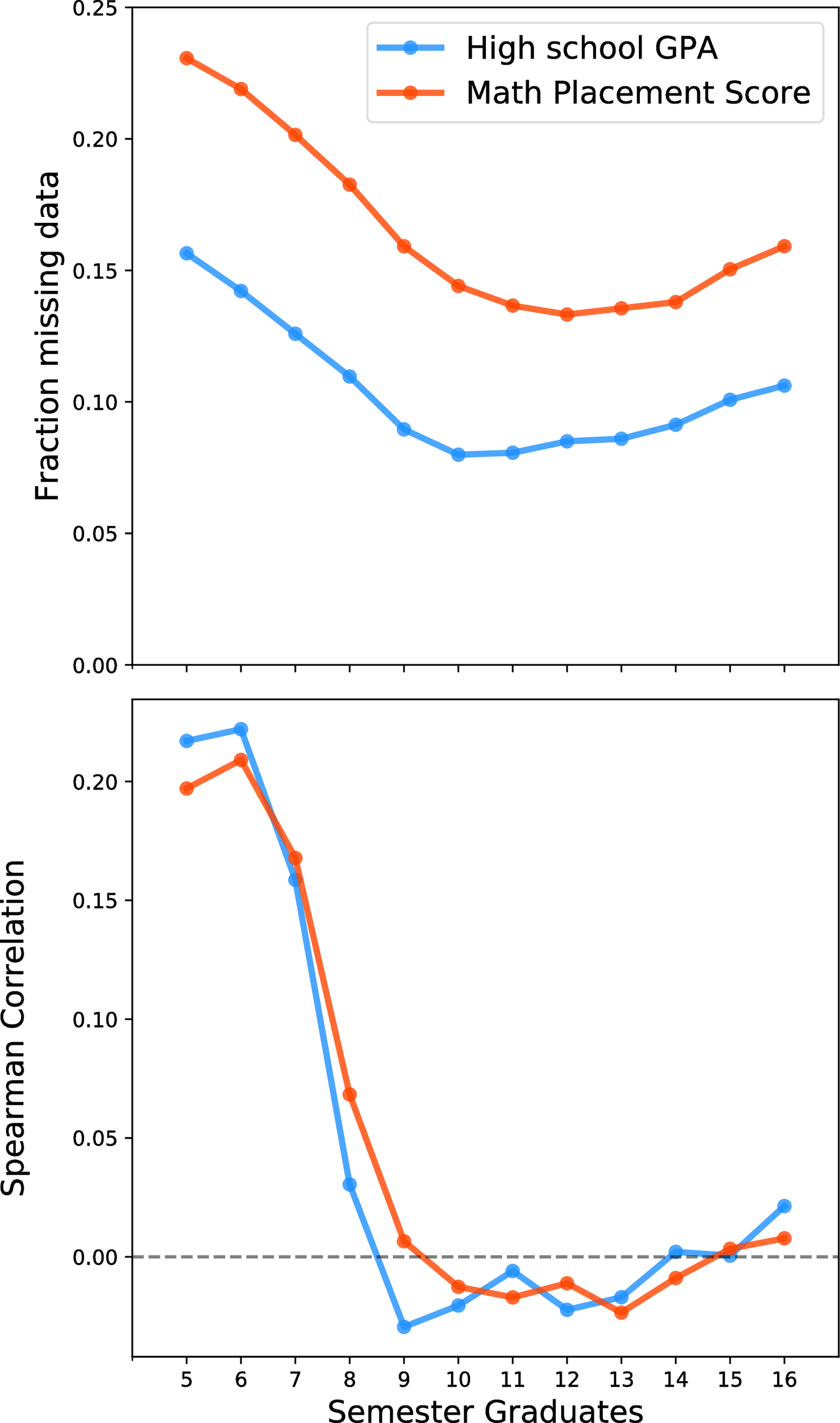

Supplement: S1 Fig — There is a small correlation in the early semesters (5-7) between whether students are graduating and if they have missing data or not. (TIF) [file pone.0242334.s001.tif]
